# Pulmonary Innate Immune Response Determines the Outcome of Inflammation During Pneumonia and Sepsis-Associated Acute Lung Injury

**DOI:** 10.3389/fimmu.2020.01722

**Published:** 2020-08-04

**Authors:** Vijay Kumar

**Affiliations:** ^1^Children's Health Queensland Clinical Unit, Faculty of Medicine, School of Clinical Medicine, Mater Research, University of Queensland, Brisbane, QLD, Australia; ^2^Faculty of Medicine, School of Biomedical Sciences, University of Queensland, Brisbane, QLD, Australia

**Keywords:** pneumonia, sepsis, ALI, ILCs, neutrophils, macrophages

## Abstract

The lung is a primary organ for gas exchange in mammals that represents the largest epithelial surface in direct contact with the external environment. It also serves as a crucial immune organ, which harbors both innate and adaptive immune cells to induce a potent immune response. Due to its direct contact with the outer environment, the lung serves as a primary target organ for many airborne pathogens, toxicants (aerosols), and allergens causing pneumonia, acute respiratory distress syndrome (ARDS), and acute lung injury or inflammation (ALI). The current review describes the immunological mechanisms responsible for bacterial pneumonia and sepsis-induced ALI. It highlights the immunological differences for the severity of bacterial sepsis-induced ALI as compared to the pneumonia-associated ALI. The immune-based differences between the Gram-positive and Gram-negative bacteria-induced pneumonia show different mechanisms to induce ALI. The role of pulmonary epithelial cells (PECs), alveolar macrophages (AMs), innate lymphoid cells (ILCs), and different pattern-recognition receptors (PRRs, including Toll-like receptors (TLRs) and inflammasome proteins) in neutrophil infiltration and ALI induction have been described during pneumonia and sepsis-induced ALI. Also, the resolution of inflammation is frequently observed during ALI associated with pneumonia, whereas sepsis-associated ALI lacks it. Hence, the review mainly describes the different immune mechanisms responsible for pneumonia and sepsis-induced ALI. The differences in immune response depending on the causal pathogen (Gram-positive or Gram-negative bacteria) associated pneumonia or sepsis-induced ALI should be taken in mind specific immune-based therapeutics.

## Introduction

Lungs serve as vital organs for the gaseous exchange in the vertebrates. They have evolved from their very primitive stage (air sacs found in the very primitive and well-armored fossil placoderm fish Bothriolepis) to the most advanced form present in mammals depending on their habitat and the oxygen demand. Thus, due to continuous gaseous exchange function, lungs serve as a very easy target organ for the airborne pathogens, allergens, and other toxicants to cause pulmonary infections or inflammation. However, pulmonary damage may be acute, or chronic depending on the intensity and the duration of the exposure. For example, chronic obstructive pulmonary disease (COPD) and allergic asthma cause chronic inflammatory changes in the lungs. Whereas, acute microbial (bacterial or viral) infections responsible for pneumonia or sepsis cause severe inflammatory damage to the lungs, leading to the development of acute lung injury/inflammation (ALI) or acute respiratory distress syndrome (ARDS) in critically ill patients (1).

ALI in response to the severe pulmonary microbial infections occurs as a result of the immunological recognition of the pathogen responsible for inducing a pro-inflammatory immune response. The ALI causes severe tissue damage, and in severe cases, irreversible pulmonary damage may lead to death. For example, the protein-rich hydrostatic pulmonary edema characterizing ALI causes refractory hypoxemia, stiffening of the lungs, and difficulty to respire. Rene Laennec (invented the stethoscope in 1861) in 1821 first described the ARDS as an “idiopathic pulmonary edema” occurring without heart failure, which was further modified into “wet lung or shock lung” (2, 3). However, Ashbaugh et al. for the first time, coined the term ARDS to describe the rapid onset of tachypnoea, hypoxemia, and the loss of compliance after a variety of stimuli (4). Sepsis is a leading (6–42%) cause of the ALI (5). Depending on the ALI/ARDS cause, age, and sex of the host, the pulmonary innate immune system plays a very significant role in the ALI pathogenesis (6).

The innate immune system serves as the first line of defense against foreign pathogens via recognizing their pathogen-associated molecular patterns (PAMPs) or microbe-associated molecular patterns (MAMPs). Also, innate immune cells recognize the damage or danger-associated molecular patterns (DAMPs) generated during the pro-inflammatory conditions disturbing immune homeostasis (7). The recognition of PAMPs or MAMPs and DAMPs involves several pattern recognition receptors (PRRs), including toll-like receptors (TLRs) and multiple germ line encoded receptors [NOD-like receptors (NLRs), retinoic acid inducible gene I (RIG-I)-like receptors (RLRs), C-type lectin receptors (CLRs) and multiple intracellular DNA sensors expressed (cGAS-STING signaling pathway, Aim 2 like receptors (ALRs)] (8–11). This induces the pro-inflammatory immune response generating different cytokines, chemokines, interferons (IFNs), and other molecules, including reactive oxygen or nitrogen species (ROS or RNS) for clearing the infection to maintain the immune homeostasis. However, the innate immune response dysregulation during infection may increase its severity via increasing the pathogen load due to the inefficient pathogen clearance or by causing increased and irreversible organ damage in patients succumbed to sepsis (12, 13). Hence, a regulated innate immune response during both acute and chronic infections is essential for clearing the infection.

The organ-specific innate immune response determines infection severity. For example, the potent innate immune response generation in the lungs during localized pulmonary infections (pneumonia) or its dysregulation as seen in the non-pulmonary sepsis (sepsis originating from other sources, including the peritoneum, urinary tract, various soft tissues, and skin)-associated acute ALI or ARDS plays a crucial role in the disease outcome (12). Thus, the major aim of the present article is to describe the pulmonary innate immune response responsible for the ALI observed during bacterial pneumonia and sepsis, as evidenced by both animal and human findings.

## Lung as an Innate Immune Organ

Lungs are the vital organs designed not only for the gaseous exchange but also serve as a major immune organ to protect the host from diseases caused by the pathogen inhalation during respiration along with allergens and xenobiotics (allergic asthma, pneumonia, sepsis-associated ALI) (12, 14). In the early 1960s, Askonas and Humphrey showed upon intravenous injection of pneumococcal antigens lungs potentially contribute to developing more specific antibodies in comparison to the rest of the lymphoid organs (15). Later on, another study in rabbits showed that the local intranasal instillation of pneumococcal antigen-induced the specific immunity and pulmonary resistance to the infection without generating the antibody (Ab)-mediated systemic immunity (16). Furthermore, the pulmonary DNA vaccine-based immunization also induces the local CD8^+^T cell-based protective anti-viral (vaccinia and influenza virus) immunity without recruiting peripheral T cells (17). This pulmonary immune response during vaccination can further be enhanced by the nasal administration of the adjuvants (18). However, the pulmonary challenge with recombinant vaccines has the potential to generate local (lung) as well as systemic immunity against pathogens (19). Thus, lung can induce protective immunity against respiratory pathogens without the involvement or activation of peripheral or systemic immunity by working as a potent immune organ.

**Figure 1:**
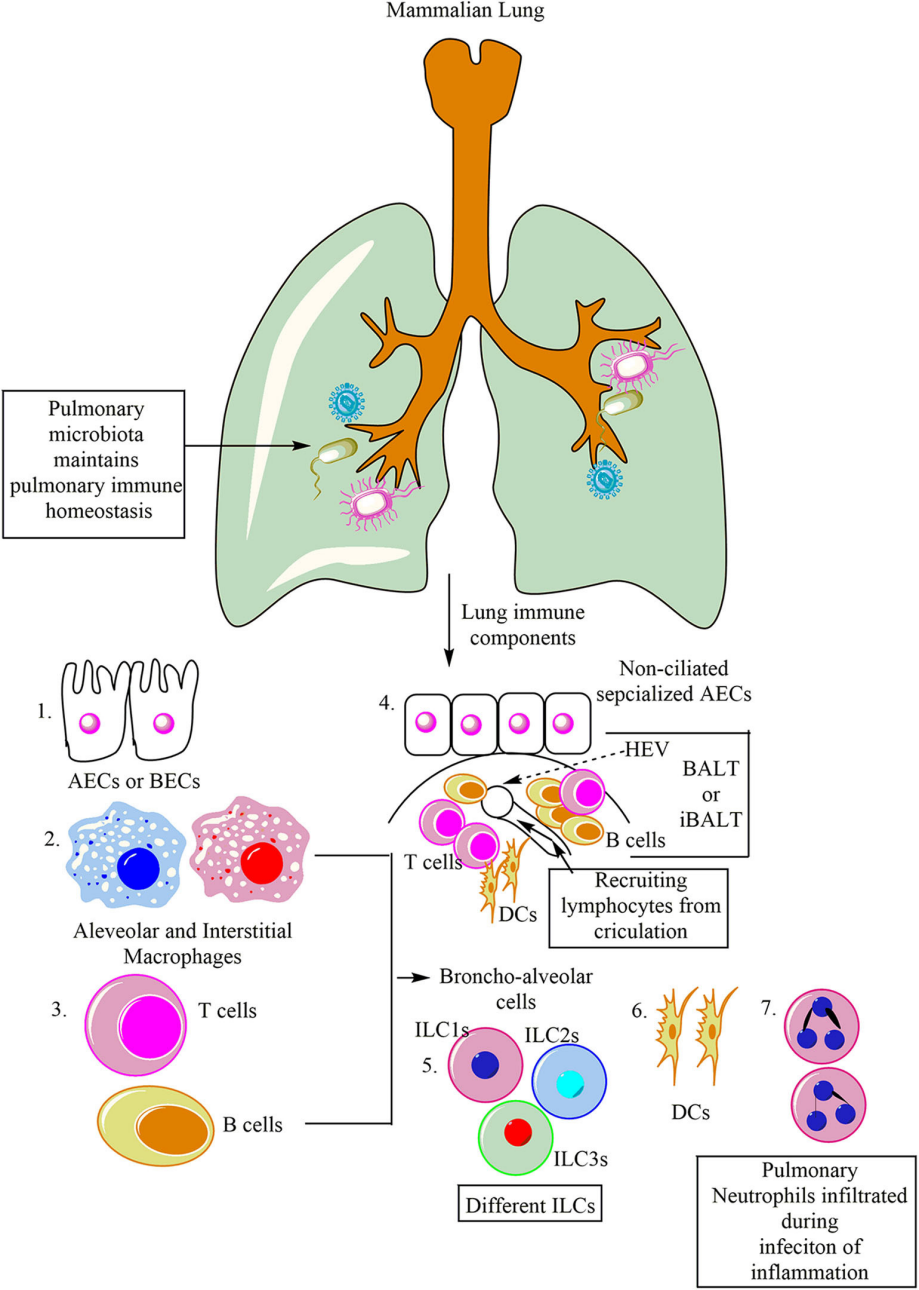
Major immune cells in the mammalian lung. Lungs are potent immune organs and contain macrophages, which may be divided into alveolar macrophages (AM) and interstitial macrophages (IMs), alveolar and bronchial epithelial cells (AECs and BECs), DCs, NK cells along with other ILCs (ILC1s, ILC2s, and ILC3s), and adaptive immune cells (different T and B cells). Neutrophils also migrate to the lungs in response to the infection or inflammatory insult. Additionally, like Peyer's patches (PPs) of gut-associated lymphoid tissue (GALT), lungs also have BALT. BALT contains T cells (T cell Zone), B cells (B cell zone), and DCs. The BALT induced in response to the infection is called iBALT.

Lungs can be categorized into two components both from a physiological and immunological point of view, (1) Upper respiratory tract serving as mucosal (IgA serves as predominating class of antibody) and glandular component, and (2) peripheral airways without any mucosal tissue (dominated by IgG antibody). Furthermore, the peripheral airways on the luminal side constantly remain in contact with the Broncho-alveolar cells (BACs, 90% of which under normal homeostasis comprise of alveolar macrophages), and 10% of which is comprised of lymphocytes (Figure 1). Thus, the pulmonary immune system is separable into different compartments, which have the potential to interact (20). Similar to the epithelial lymphocyte compartment of the gut, a compartment of lymphocytes residing in the respiratory tract epithelium over the epithelial membrane and between the epithelial cells also exists. Thus, protecting the host from invasive pulmonary infections. The other compartment of the respiratory lymphoid cells (RLCs) comprises of the organized lymphoid tissues lying within the bronchial walls. This RLC compartment comprises of either solitary lymphoid follicles (SLFs) or their aggregates resembling the Peyer's Patches (PPs) of the intestine (21, 22). Thus, this bronchus-associated lymphoid tissue (BALT) is morphologically and functionally analogous to the gut-associated lymphoid tissue (GALT) of the intestine (23, 24). For example, receptor activator of nuclear factor-κB and its ligand (RANKL) is a common inducer of M cells in the lungs and gastrointestinal tracts (GITs) (25). M cells play a crucial role in respiratory diseases (26).

The BALT is covered by a lymphoepithelium, and its follicle-associated epithelium selectively samples both soluble and particulate matter from the respiratory tract lumen (Figure 1) (27, 28). Of note, in humans, BALT is present only in the lungs of kids and adolescents, and adults show BALT only during chronic inflammatory diseases, where it is called inducible BALT (iBALT) (Figure 1) (29). On the other hand, BALT may present in the fetal and neonate's lungs, depending on the antigenic stimulation (30, 31). However, these RLCs comprising the lymphoid follicles of the BALT in humans expand or proliferate considerably in a group of patients suffering from recurrent respiratory tract infections (RTIs) of unknown etiology due to the occlusion of the bronchiolar or bronchial lumen (32). B cells are the major immune cell population of the BALT responsible to generate IgA (Figure 1) (20, 33). T cells comprising T cell zone are also present in BALT. T cell zones also have DCs. BALTs also have high endothelial venule (HEV), which serves to transport lymphocytes and antigens to and from the circulation (Figure 1). The IgA produced may bind to the lymphocytes to increase their Ab-dependent cytotoxic action. The secreted IgA also protects against viral and bacterial infections along with the allergy. The other compartment comprises of BACs, which can be obtained through broncho-alveolar lavage fluid (BALF) from the peripheral airways. BALF may contain alveolar macrophages (AMs), innate lymphoid cells (ILCs), and dendritic cells (DCs), providing protection against pathogens, toxicants, and allergens inhaled. These pulmonary innate immune cells serve as antigen-presenting cells (APCs) and secrete several cytokines and chemokines to regulate both the pulmonary innate and adaptive immunity. Under normal healthy conditions, BACs in BALF mainly comprises of AM (90%) and rest (10%) are lymphocytes (14). These lymphocytes, via lymph, circulate through the lung and patrol for potential antigen inhaled or entered into the lung through circulation.

The pulmonary immune system matures in the postnatal environment depending on the richness and the type of antigen exposure to the host (34). However, during *in utero* embryonic development lungs remain sterile, but during vaginal delivery, they acquire maternal microbiota (35, 36). The pulmonary microbiota helps in the pulmonary immune system development, tolerance induction, and its homeostasis (Figure 1) (37, 38). The pulmonary residential epithelial cells, ILCs, and AMs along with other pulmonary immune cells, are essential to maintain the steady-state in the lungs. However, their ability to recognize different airway pathogens and allergens also induces inflammatory changes in the lungs. Under some situations, these pulmonary inflammatory changes are mild and resolve by itself, but the ALI observed during bacterial pneumonia and sepsis may prove harmful to the host depending on the severity of the infection and the inflammatory innate immune response.

## Pulmonary Innate Immune Response During Bacterial Pneumonia

According to the National Center for Health Statistics, bacterial pneumonia and influenza comprised eighth causes of mortality in the United States in 2014–2018 (39, 40). However, in children, among infectious diseases, pneumonia is the single most cause of death all over the world (41). Thus, pneumonia is a serious life-threatening infection among the children and older population. Pneumonia pathogenesis is a very complex process involving the microbial invasion of the lower respiratory tract through community or hospital spread. It may occur through inhalation of the causal pathogen. For example, *S. pneumoniae* is the most common pathogen responsible for community-acquired pneumonia (CAP). In addition to the *S. pneumoniae, Legionella pneumophila, Mycoplasma pneumoniae, Chlamydophila pneumoniae, Chlamydophila psittaci*, and *Coxiella burnetii* are several other common pathogens responsible for CAP (42, 43). Most hospital-acquired pneumonia are caused by Gram-negative pathogens (*Klebsiella pneumoniae, Pseudomonas aeruginosa*, etc.). The details of CAP and hospital-acquired pneumonia (HAP) are described somewhere else (41). The pulmonary innate immune response during pneumonia initiates with the activation of residential innate immune cells (AECs, AMs, etc.) inducing the neutrophil infiltration into the lungs. Toll-like receptor 4 (TLR4) activation induced immune response protects the experimental animals infected with Gram-positive (*Streptococcus pneumoniae*) or Gram-negative bacteria (*Klebsiella pneumoniae*) induced pneumonia (44).

### Neutrophil Infiltration in Lungs During Pneumonia-Associated ALI

The mechanism of neutrophil infiltration in the lungs varies from the process involved in other organs and has been described in detail somewhere else (45, 46). The mechanism of neutrophil infiltration in the lungs during ALI varies during Gram-negative and -positive bacterial pneumonia (47). For example, during Gram-negative bacterial (*E. coli* or *P. aeruginosa*) pneumonia, alveolar neutrophil infiltration is mediated by CD18 or β2 integrin, whereas in Gram-positive bacterial (*S. pneumoniae*) pneumonia, it is mediated by the CD29 or β1 integrin (48). Additionally, patients with ALI show an elevated chemokine (CXCL8 or IL-8, CXCL1, or keratinocyte-cell derived chemokine (KC), CXCL5, or epithelial cell-derived neutrophil-activating peptide-78 (ENA-78), and CCL-2) levels in their BALF, which further regulate neutrophil infiltration into the lungs (49, 50).

**Figure 2:**
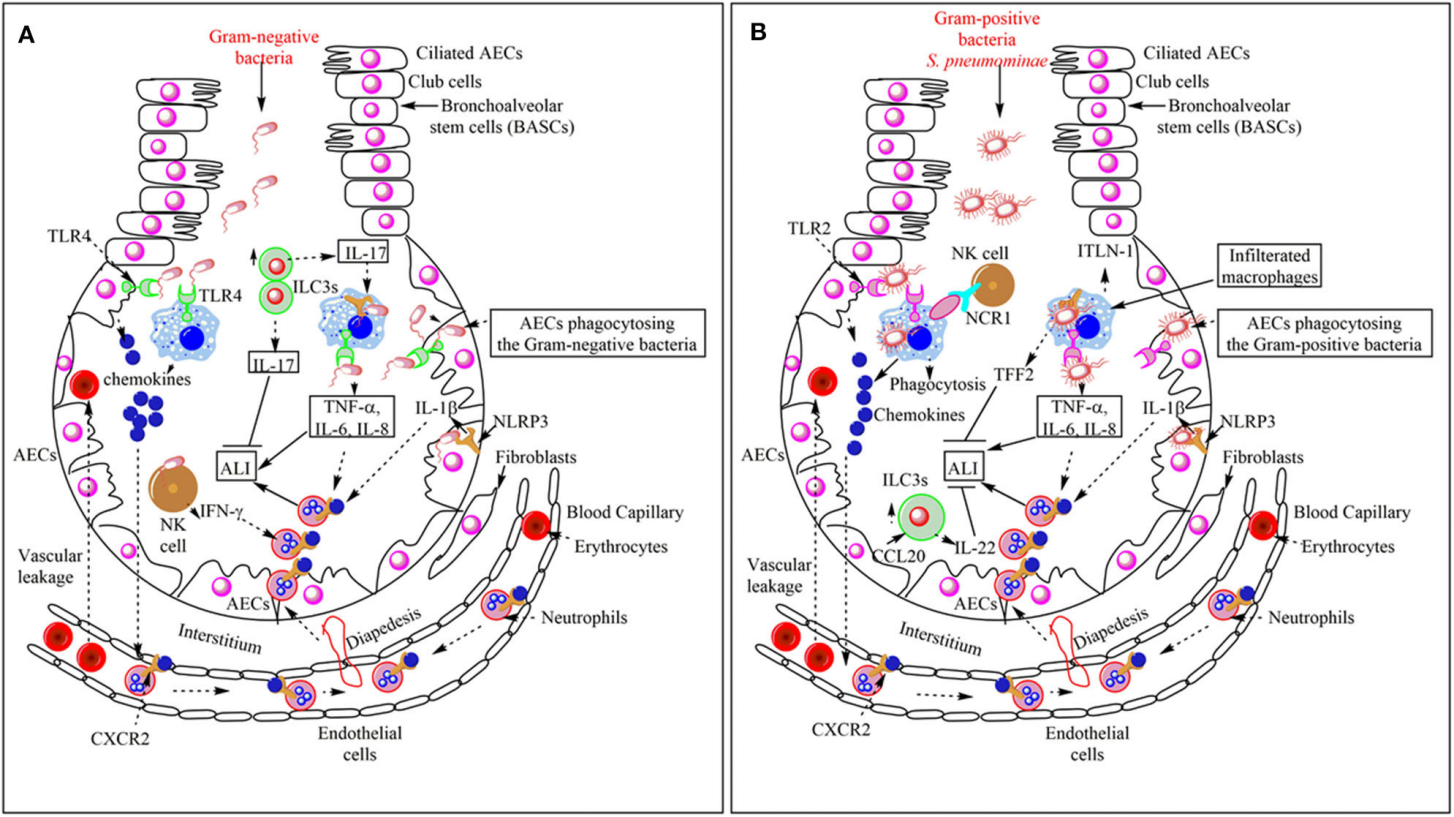
Overview of the bacterial pneumonia-associated innate immune response responsible for ALI. **(A)** Gram-negative bacterial pneumonia and ALI. The PRRs [TLRs (TLR4) and Inflammasome proteins (NLRP3)] expressed on the pulmonary innate immune cells (AECs, BECs, AMs, DCs, and NK cells) recognize Gram-negative bacteria in the lungs. This recognition induces pro-inflammatory cytokine (TNF-α, IL-6, IL-8, and IL-1β) and chemokine release. Chemokines and pro-inflammatory cytokines induce the neutrophil infiltration in the alveoli from the pulmonary blood capillaries through their vascular endothelium via diapedesis. The infiltrated neutrophils help in the pathogen clearance but also cause bystander inflammatory pulmonary tissue damage via damaging PECs or AECs. In the severe pneumonia-associated ALI, the vascular leakage of proteins and erythrocytes also occurs in the lungs. The pulmonary NK cells also release IFN-γ, which further enhances neutrophil infiltration and ALI. However, the increase in ILC3s at later stages increases the IL-17 level. This cytokine helps in the increased phagocytosis of the pathogen and the resolution of the Gram-negative bacterial pneumonia-induced ALI. **(B)** Gram-positive bacterial pneumonia-associated ALI. The recognition of the Gram-positive bacteria (*S. aureus*) induces the increased the production of chemokines and pro-inflammatory cytokines (TNF-α, IL-1β, IL-6, and IL-8), which via binding to the corresponding receptors (CXCR2) on the neutrophils induce their diapedesis to the lung alveoli from the pulmonary vascular endothelium. Neutrophils, along with bacterial clearance, also cause ALI. Pulmonary vascular endothelium damage also causes vascular leakage. The Pulmonary NK cells via their NCR1 interact with AMs to further increase the pro-inflammatory cytokine release, which further aggravates the neutrophil infiltration, pathogen clearance, and ALI also. The NLRP3 also works independently of inflammasome activation via inducing the release of TFF2 and ITLN-1. TFF2 inhibits ALI and helps in its resolution, whereas ITLN-1 clears the infection via increasing the pathogen phagocytosis. The CCL20 released from AECs increases the pulmonary ILC3 numbers, which release IL-22 that inhibits ALI and helps in its resolution.

The CXCR2 (a chemokine receptor) binding to different chemokines [CXCL1, CXCL8 (in humans), CXCL5, CXCL2, CXCL3, CXCL6, and CXCL7 (in humans)] regulates the neutrophil infiltration in the lungs (Figure 2A) (51). The lung epithelial cells (LECs) produce the CXCL5 during bacterial (*E. coli*) pneumonia that induces neutrophil infiltration in the lungs, whereas in naïve murine blood, platelets are a crucial source of CXCL5 (51). However, CXCL5 deficiency during *E. coli* pneumonia increases neutrophil influx in the lungs, accelerates the pathogen clearance, improves pulmonary edema, and protects the mice from severe pneumonia and, thus, the ALI (52). CXCL5^−/−^ mice do not show much decrease in CXCR2 expression on bone marrow and blood neutrophils as compared to the wild type (WT) mice upon *E. coli*-induced pneumonia, but the CXCR2 expression on neutrophils remains unchanged during intranasal lipopolysaccharide (LPS) challenge (52). In the absence of CXCL5, CXCL1, and CXCL2 bind to the Duffy Antigen Receptor for Chemokines (DARC) to increase the neutrophil infiltration in the lungs, which enhances bacterial clearance, and protects the animal from severe pneumonia (52). The levels of these chemokines (CXCL8) affect the severity of ALI and the mortality among patients regulating neutrophil infiltration (53). The CXCL1 regulates neutrophil infiltration and the bacterial clearance during *K. pneumoniae*-induced pneumonia via regulating the CXCL2/MIP-2 and CXCL5, and NF-κB and MAPKs activation in the lungs (54). Thus, the pulmonary neutrophil infiltration is crucial in the ALI induction and its resolution (55, 56). The pulmonary residential innate immune cells [Airway epithelial cells (AECs), macrophages, dendritic cells (DCs), and innate lymphoid cells (ILCs)] are crucial in the pathogenesis of bacterial pneumonia and associated ALI and its outcome.

### Airway Epithelial Cells (AECs) and PRRs (TLRs and Inflammasomes) During Pneumonia

AECs comprising of bronchial epithelial cells (BECs) and alveolar epithelial cells [categorized into (1) type I AECs, which are primarily involved in facilitating gaseous exchange and may also recognize pathogens, and (2) type II AECs, also called type II pneumocytes and serve as innate immune cells] serve as a protective mechanical barrier against inhaled pathogens responsible for pneumonia (57, 58). Type II pneumocytes also secrete surfactant proteins on their apical side. These surfactant proteins serve as mucins. The type II AECs secrete repair enzymes (fibrinogen or FBG) basolaterally, which respond to the change in osmotic pressure of the cell very quickly and sense pore-forming toxins secreted by pathogenic bacteria (59). The released FBG helps in the cellular response in response to the inflammatory cell damage (60). AECs also serve as potent regulators of the generation of the primary immune response against invading pathogens via releasing various immune mediators [antimicrobial peptides (AMPs), cytokines] and interacting directly with other immune cells (macrophages, neutrophils, and DCs) (61). The prolonged activation of AECs may prove harmful to the host due to the release of large quantities of pro-inflammatory cytokines, chemokines, and their increased cell death (necrosis or necroptosis).

During pneumonia, AECs recognize various pathogens due to the expression of different PRRs, including TLRs [TLR1, TLR2, TLR4, TLR5, TLR6 (Extracellular TLRs), and TLR3, TLR7, TLR8, and TLR9 (Intracellular TLRs)] and NLRs comprising inflammasome (58, 62–64). The recognition of pathogens by these PRRs serves as the first line of defense and helps in the pathogen clearance. The downstream adaptor molecules [myeloid differentiation primary response protein 88 (MyD88) and Toll/IL-1R domain-containing adaptor-inducing IFN-β (TRIF)] of TLR signaling play crucial roles in bacterial pneumonia. For example, MyD88^−/−^ and TRIF^−/−^ mice develop severe pneumonia due to the profound bacterial growth during Gram-negative pneumonia (*K. pneumoniae, P. aeruginosa*, and *E. coli*) in response to the impaired immune response, including the reduced generation of Th1 immune response (TNF-α, IL-6, and IL-8) and almost no neutrophil influx and regulated upon activation normal T Cells expressed and presumably Secreted (RANTES or CCL5) production (65–68).

The TRIF signaling in response to the TLR activation during *K. pneumoniae*-induced pneumonia also exerts antibacterial defense via inducing the interferon (IFN)-x03B3 in the lungs (69). However, Toll/IL-1R Domain-Containing Adaptor Protein (TIRAP) plays a critical role during *K. pneumoniae*-induced pneumonia but not during *P. aeruginosa*-induced pneumonia due to the attenuation of neutrophil sequestration, and MIP-2, TNF-α, IL-6, and LIX (lipopolysaccharide-induced CXC chemokine) production (70). The LIX production, neutrophil infiltration, and bacterial clearance during *P. aeruginosa*-induced pneumonia do not require TIRAP (70). The TLR2-induced MyD88 activation is not required for the *S. aureus* clearance during pneumonia and only exerts the potent inflammatory immune response, but it plays a crucial role in *P. aeruginosa* clearance (71). Thus, TLR2 activation does not have a significant role in the pathogen clearance and survival of the mice but is only required for the inflammatory immune response during Gram-positive bacterial (*S. pneumoniae*) pneumonia (Figure 2B) (72). The TLR2 signaling activation during Gram-positive bacterial (*S. pneumoniae*) pneumonia increases the non-small cell lung cancer cell (NSCLC) metastasis (73). Thus, Gram-positive bacterial pneumonia may increase the metastasis of cancer cells in cancer patients.

Inflammasomes and their component proteins also play a crucial role in pathogen detection and clearance during pneumonia. For example, NLRP1 (NLR Family Pyrin Domain Containing 1) enhances the host's resistance to pneumonia via detecting their virulence factors [*Bacillus anthracis* lethal factor (LF) protease] (74). The LF protease induces the proteasome-mediated degradation of amino-terminal domains of NLRPB1 to liberate the carboxyl-terminal fragment, a potent caspase-1 (CASP1) activator (75). Also, the NLR Family Pyrin Domain Containing 3 (NLRP3) activation in BECs during various pneumonia-causing bacterial (*K. pneumoniae, S. pneumoniae, S. aureus, C. pneumoniae*, and *L. pneumophila*) infections protects the host from infections (Figures 2A,B) (63, 76, 77). The human BECs also express NLRP3 inflammasome (78). The NLRP3-mediated control of *K. pneumoniae*-induced pneumonia involves the increased neutrophil infiltration, macrophage necrosis, and the release of high-mobility group box-1 protein (HMGB-1) (Figure 2A) (79).

The apoptosis-associated speck-like protein containing a caspase activation and recruitment domain (CARD) or pyrin domain (PYD) (ASC, also known as PYCARD) is an inflammasome adapter protein required for the formation of the absent in melanoma 2 (AIM2) and NLRP3 inflammasomes. Inflammasome activation causes the ASC speck formation, which forms a platform to activate caspase-1 (CASP-1). However, NLRP3 and ASC maintain pulmonary innate immune homeostasis during *S. pneumoniae*-induced pneumonia through an inflammasome independent manner without activating the CASP1 and CASP11 (80). During this process, they (NLRP3 and ASC) stimulate the optimal expression of several mucosal innate immune proteins, including trefoil factor 2 (TFF2) and intelectin-1 (ITLN-1, a secretory galactofuranose-binding lectin) via expressing the SAM pointed domain-containing Ets transcription factor (SPDEF), which facilitates the mucosal defense factor genes (Figure 2B) (80). SPDEF activation involves STAT6 activation. TFF2 protects from increased inflammatory damage via inducing decreased neutrophil recruitment through inhibiting the endothelial vascular cell adhesion molecule 1 (VCAM1) expression and nitric oxide (NO^.^) release from macrophages via inhibiting inducible nitric oxide synthase (iNOS) (81, 82). TFF2 also antagonizes IL-12 (a cytokine required for inducing IFN-γ production and activating Th1 immune cells) secretion from dendritic cells (DCs) and macrophages (83). TFF2 also serves as mucosal healers via protecting mucosal damage, promoting cell motility, and alveolar type 2 cell proliferation, and restores pulmonary gas exchange after infection (84, 85). TFF2 also induces IL-25 and IL-33 after infection to induce type 2 immunity and repair (86). Pulmonary macrophages also utilize the TFF2/Wnt axis to induce pulmonary epithelial cell proliferation to repair the damage following ALI (87). ITLN-1 provides protection via directly binding to the *S. pneumoniae* and representing them to phagocytes for phagocytosis (Figure 2B) (80, 87). The BECs express ITLN-1 and may also clear the *S. pneumoniae* via phagocytosis (88–90).

Aged mice exhibit a reduced NLRP3 expression and function, which increases their susceptibility to developing pneumonia, ALI, and mortality (91). The lower expression and function of NLRP3 in aged immune cells (macrophages, epithelial cells, and DCs) attribute to the increased unfolded protein responses (UPRs), which causes a decreased inflammasome assembly and function increasing the severity of pneumonia caused by *S. pneumoniae* (92). The aging also increases the susceptibility of the host to secondary *S. pneumoniae*-induced pneumonia due to the decreased NLRP3 expression and function in the aged lung (93). The treatment of these aged mice with inflammasome activators [Nigericin, which promotes potassium (K^+^) efflux increases the synthesis and release of inflammasome activation-dependent cytokines (IL-1β and IL-18)] increase their survival and decreases their susceptibility toward pneumonia and ALI. Furthermore, the pre-treatment of aged mice with endoplasmic reticulum (ER) chaperone and the stress-reducing agent tauroursodeoxycholic acid (TUDCA) decreases the pneumonia-associated mortality among the aged mice due to the activation of the NLRP3 inflammasome, which increases the pathogen clearance, and lowers the infection-associated pneumonitis (92). The aged mice also express lower levels of TLR1, TLR6, and TLR9 in the lungs, which also increases their susceptibility to pneumonia (93).

Of note, during lethal pneumonia caused by a low dose of serotype 3 *S. pneumoniae*, NLRP3 increases the incidence of ALI and mortality due to the bacterial dissemination and the development of the sepsis (94). Also, during *S. aureus*-induced pneumonia, NLRP3 deficiency prevents the onset of severe necrotic pneumonia via promoting bacterial clearance (95). The NLRP3 activation by α-hemolysin during *S. aureus* pneumonia induces necrotic pulmonary injury or necrotizing pneumonia independent of IL-1β signaling (95, 96). The NLRP3 activation by α-hemolysin in innate immune cells depends on A Disintegrin and metalloproteinase domain-containing protein 10 (ADAM10) expression and activity (97). ADAM10 binding with α-hemolysin increases NLRP3 activation and cell death due to the availability of ADAM10 on the cell surface. However, ADAM10 protease activity does not play a significant role in NLRP3 activation. Thus, the profound NLRP3 inflammasome activation depending on the severity of the infection proves harmful to the host. In addition to the NLRP3 inflammasome, *S. aureus* pneumonia also activates NLRC4 inflammasome to induce necroptosis through inhibiting the IL-17A-induced neutrophil accumulation in the lungs and IL-18 production (98). The deficiency of NLRC4 increases the pulmonary neutrophil infiltration, decreases the necroptosis, increases the pathogen clearance, and improves the host survival. Thus, the loss of NLRC4 in both hematopoietic and non-hematopoietic cells protects the host against *S. aureus* pneumonia (98).

The murine AECs also express NLRP6 inflammasome (63). NLRP6 activation during *S. aureus* pneumonia also increases the pyroptosis and necroptosis, causing necrotizing pneumonia, increases bacterial burden in the lungs, and decreases the pulmonary neutrophil infiltration (99). The neutrophils isolated from NLRP6 knockout (KO) animals exhibit an increased NADPH-dependent reactive oxygen species (ROS) production and increased bacterial killing. Thus, therapeutic targeting of NLRP3, NLRC4, and NLRP4 inflammasome during Gram-positive bacteria-induced severe pneumonia responsible for ALI may prove beneficial to the host. An experimental study has shown the beneficial effects (inhibition of ALI, decrease in pro-inflammatory cytokines levels, and decrease in the mortality) of resveratrol during *K. pneumoniae*-induced pneumonia through the NLRP3 inflammasome inhibition (100). NLRC4 activation during Gram-negative bacterial (*K. pneumoniae, P. aeruginosa*) pneumonia proves beneficial to the host via producing IL-1β, IL-17A, and neutrophil chemoattractants (keratinocyte cell-derived chemokines, MIP-2, and LPS-induced CXC chemokines) in the lungs (101). However, during *P. aeruginosa* pneumonia, NLRC4 activation-induces inflammatory lung damage, increases pulmonary bacterial burden, and necroptosis (102). Hence the inhibition of NLRC4 inflammasome activation during Gram-negative pneumonia remains a tricky scenario, and further studies will prove helpful in the direction.

The TLR2, TLR4, and MyD88 deficiency did not alter the host response during lethal pneumonia. Any abnormality in the AEC function may lead to the predisposition of the host toward pulmonary infections, including bacterial pneumonia due to the enhanced microbial colonization. For example, patients with allergic asthma are more prone to develop bacterial pneumonia due to the increased pathogenic bacterial colonization, including *Staphylococcus aureus* (*S. aureus*). It may be explained as the higher Th2 cytokines (IL-4 and IL-13) decrease the antimicrobial action of AECs via suppressing the synthesis of human β-defensins 2. However, during streptococcal or pneumococcal pneumonia, AECs also express secreted and transmembrane (Sectm) 1, Sectm1a, and Sectm1b genes due to the type 1 IFN signaling induction in AECs via signal transducer and activator of transcription 1 (STAT-1) activation (103). The Sectm1a binds to the neutrophils only in the presence of the infection and increases the CXCL2 expression. Thus, Sectm1 synthesis and release by AECs during pneumonia increase the neutrophil infiltration into the lungs and helps to clear the infection. However, its dysregulated synthesis may lead to the development of ALI or ARDS.

PECs protect from *K. pneumoniae*-induced pneumonia via ingesting and controlling their number through phagocytosing them via producing the complement component C3, which opsonizes them for phagocytosis (Figure 2A) (104). CD46 recognizes the C3 opsonized *K. pneumoniae* for the AEC-mediated phagocytosis or internalization (105). However, the complement resistant strains of *K. pneumoniae* have been emerged and are posing a potential threat to the host (106). The type 1 AECs also highly express epithelial membrane protein 2 (EMP2), a tetraspan protein, which promotes recruitment of different integrins (α6β1, αVβ3) and adhesion molecules (ICAM-1) to the lipid rafts (107). Both rodent and human type II AECs and AMs do not express EMP2 (108). The EMP2 expression of type 1 AECs plays a crucial role in the transepithelial neutrophil migration into the alveoli by regulating the expression of integrins and adhesion molecules (ICAM-1) and suppression of caveolins during bacterial pneumonia (109). Mice lacking EMP2 show a decreased neutrophil infiltration in the alveoli and lung injury during pneumonia. Thus, the activation of the residential PECs as innate immune cells during bacterial pneumonia plays a crucial role in the pathogenesis of pneumonia-associated ALI and its outcome depending on the pathogens (Gram-positive or Gram-negative bacteria) causing pneumonia and the associated immune response.

### Pulmonary Innate Lymphoid Cells (ILCs) During Pneumonia and Associated ALI

ILCs serve as immunoregulatory innate immune cells at mucosal surfaces and play a crucial role in the pathogenesis of inflammation and inflammatory diseases (110, 111). The details of their development, classification, regulatory transcription factor (TFs), and function are described somewhere else (112–114). ILCs are divided into three major categories depending on their effector functions and transcriptional requirements: (1) Group 1 ILCs include type 1 ILCs, and Natural Killer (NK) cells, (2) Group 2 ILCs or ILC2s, and (3) Group 3 ILCs or ILC3s (114). Group 1 ILCs, including NK cells, are a rapid source of interferon-γ (IFN-γ), and mice deficient in IFN-γ develop more severe *K. pneumoniae* or *L. pneumophila*-induced pneumonia upon intratracheal inoculation of the pathogen due to impaired IL-1 and IL-6 production, and the defective clearance of the bacteria (115, 116).

NK cells in the lungs are present in its parenchyma in humans and comprise 10–20% of total lung lymphocytes, and in mice, they account for 10% of total lung lymphocytes (117, 118). Human lung NK cells are mostly CD16^+^CD56^low^, and KIR^+^CD57^+^NKG2A^−^ highly differentiated NK cells are also found in the lungs (117, 119). However, the pulmonary resident NK cells express CD69, CD49a, and CD103, and most of them are CD56^high^CD16^−^ and display a lesser mature form (120). In mice, pulmonary NK cells protect against *K. pneumoniae*-induced pneumonia via secreting IFN-γ and IL-22, which launch the bacterial growth-controlling interactions between alveolar macrophages and NK cells (Figure 2A) (121, 122). IFN-γ plays a crucial role in the bactericidal action of alveolar macrophages and the release of NK cell amplifying IL-12 and CXCL10 (122). The NCR1 (natural cytotoxicity receptor 1) on pulmonary NK cells controls their activation and the IFN-γ release during the early stages of *S. pneumoniae*-induced pneumonia without mediating the pathogen recognition (123). However, NCR1 ligands are expressed by pulmonary macrophages and DCs, which directly interact with NK cells during the early stages of *S. pneumoniae*-induced pneumonia (Figure 2B). This interaction increases their phagocytic activity required to clear the infection and mounting the effective immune response (Figure 2B).

Group 2 ILCs secrete Th2 cytokines [IL-4, IL-5, IL-6, IL-9, IL-13, and Amphiregulin (Arg)] and group 3 ILCs depending on the cytokine released, can be categorized into IL-17 secreting and IL-22 secreting ILC3s. In addition to these cytokines, ILC3s also secrete IL-26 (in humans), GM-CSF, and TNF-α (124). Lymphoid tissue inducer (LTi) cells also belong to group 3 ILCs and secrete IL-22 and IL-17. However, LTi cells have not been seen in the lungs in homeostasis and acute inflammation (125). In human lungs, they have also not been identified due to the lack of known human LTi markers (126). For example, CCR6 is a marker for mice LTi cells, but in humans, all ILC3s express CCR6, and therefore CCR6 does not serve as a marker for human LTi cells (127). Even tertiary lymphoid organs or follicles (TLOs or TLFs), such as iBALT form in the lung tissues of Rorc^−/−^ and Id2^−/−^ mice, which lack LTi cells, following influenza virus infection and inflammation (128). However, iBALT development depends on IL-17 secreted by Th17 cells, which triggers lymphotoxin-independent expression of CXCL13 and CCL19. Thus, LTi cells are dispensable for the aspect of lung immunity.

Lungs are the crucial sites for all the three groups of ILCs (125). Haemophilus influenza pulmonary infection increases the number of IFN-γ producing ILC1-like (Lin^−^IL-7Rα^+^IL-12Rβ2^+^IL-18Rα^+^Tbet^+^) cells and increases the pulmonary inflammatory immune response due to the plasticity among pulmonary ILC2s (129). AECs or PECs or pulmonary macrophages during pneumonia secrete IL-1β that governs the ILC2s plasticity (130). IL-1β impacts ILC2 plasticity via inducing the low expression of T-bet (TF) and inducing the IL-12Rβ2 expression, which converts these cells into ILC1s in the presence of IL-12 (131). The treatment with IL-12 during pneumonia exerts a protective action via increasing the infiltration of inflammatory cells (ILC1s, NK cells, and neutrophils) and inflammatory cytokines (IFN-γ) (132, 133). The transforming growth factor-β1 (TGF-β1) secreted by AECs or PECs primes pulmonary ILC2s (134). Pulmonary ILC2s express TGF-βRII. The CD127^+^CD90^+^CCR6^+^RORγt^+^ group 3 ILCs have been identified in the lung mucosa (135). Pulmonary ILC2s are unable to migrate efficiently within the lung tissue in the absence of TGF-β (134). IL-33 protects against pneumonia via enhancing bacterial clearance and improving the mortality via increasing the neutrophil infiltration and pulmonary ILC2s number (136). The ILC2s convert into ILC1s, which clear the pathogens. Also, these ILC2s are crucial for IL-13-dependent differentiation of pulmonary M2 macrophages, required for the resolution phase of inflammation and infection (137).

The *S. pneumoniae* pneumonia frequently induces the group 3ILCs accumulation in the lungs, which produce IL-22 to protect against severe pneumonia (Figure 2B). Furthermore, the administration of TLR5 agonist (flagellin) enhances the IL-22 production from group 3 ILCs during *S. pneumoniae*-induced pneumonia (Figure 2B). Studies have also shown earlier, the protective action of mucosal (including sublingual root) flagellin administration to mice infected with *S. pneumoniae*-induced pneumonia without the activation of NLRC4 inflammasome (138, 139). Also, the TLR5 agonist (flagellin) administration increases the efficacy of antibiotic treatment during pneumonia (140). The group 3 ILCs activation to produce IL-22 during *S. pneumoniae*-induced pneumonia also involves the activation of pulmonary dendritic cells (DC). Thus, the activation of pulmonary mucosal group 3 ILCs may prove beneficial to contain the pulmonary infection or pneumonia associated with severe lung inflammation and ALI. The number of group 3 ILCs producing IL-17 also increases during *K. pneumoniae*-induced pneumonia, which helps in the resolution of pulmonary inflammation at later stages to prevent the development of ALI during pneumonia (Figure 2A) (141). The release of TNF-α increases the pulmonary ILC3s number and also acts on AECs or PECs to synthesize CCL20. CCL20 chemoattracts ILC3s at the site of infection and inflammation (Figure 2B). Also, ILC3 produce IL-17A, which enhances the phagocytic uptake and killing of the bacteria by pulmonary macrophages to clear pneumonia (Figure 2A). Thus, ILC3s secrete IL-17A to clear infection during early stages and help in the resolution of the inflammation to prevent ALI during pneumonia.

The recruitment of IL-22 producing ILC3s into the lungs of neonate mice on exposure to commensal bacteria protects them from neonatal pneumonia (142, 143). This protection involves the intestinal mucosal DCs mediated sensing of commensal bacteria. Furthermore, the murine gut microbiota comprising segmented filamentous bacteria (Sfb) controls the resistance to the *S. aureus* pneumonia via enhancing the number of IL-22 and IL-17 producing innate immune cells (144). Hence pulmonary ILC3s protect the host from pneumonia and associated ALI during early childhood and later in adult life. Thus, pulmonary ILCs serve as crucial pulmonary innate immune cells to protect against pneumonia-induced ALI and in the resolution of the lung inflammation during pneumonia.

### Pulmonary Macrophages During Bacterial Pneumonia and Associated ALI

Pulmonary macrophages account for 90–95% of lung immune cells at homeostasis (145). They are of two types: (1) Interstitial macrophages or IMs (reside in lung parenchyma and highly express CD11b but lower levels of CD11c), and (2) Alveolar macrophages or AMs (located in airway space, express high levels of CD11c and low levels of CD11b at their quiescent stage) in the healthy lung (146). Both AMs and IMs express the macrophage-specific markers [CD64 or Fc-gamma receptor 1 (FcγRI) and MER Proto-Oncogene or Tyro-Axl-MerTK (TAM) family of receptor Tyrosine Kinase (Mertk) is involved in efferocytosis] (147, 148). AMs are crucial for maintaining pulmonary immune homeostasis and host defense due to their unique location at the interface between the pulmonary mucosa and the external environment, and are inherently suppressive, whereas IMs exhibit the regulatory function in the lung (149). IMs produce high levels of IL-10 as compared to the AMs, which mainly produce non-specific antimicrobial molecules, including NO^.^, TNF-α, and IFN-γ (146). The steady-state AMs express CD206 (a mannose receptor, which is a C-type lectin and serves as a PRR) and β-glucan specific receptor (Dectin-1), which are also expressed by alternatively-activated macrophages (AAMs) or M2 as their definitive markers (150, 151). The serum CD206 (sCD206) levels increases in the patients of community-acquired pneumonia (CAP) with the increase in its severity [pneumonia severity index (PSI)], which can be used for CAP prognosis (152). Also, the infiltration of CD206^+^ macrophages increases in the lungs of patients with fatal pneumonia.

AMs play a crucial role in the pathogenesis of bacterial pneumonia and associated ALI. For example, AMs during Gram-negative bacterial pneumonia produce TNF-α, which induces granulocyte-macrophage colony-stimulating factor (GM-CSF) in AECs that elicits proliferative signaling in AECs via autocrine stimulation contributing to the alveolar epithelial barrier restoration (153). However, during *S. pneumoniae*-induced pneumonia infiltrating peripheral macrophages replace the resident AMs and IMs. Also, the AM-mediated clearance of apoptotic cells decreases their potential to phagocytose the bacterial pathogens, which increases the bacterial burden in the lungs (Figure 2B) (154, 155). The efferocytosis induces the release of prostaglandin E2 (PGE2), which binds to the prostanoid receptors EP2-EP4 activating inhibitory cAMP and PKA pathway, which impairs the neutrophil infiltration and induces the IL-10 release to impair the pathogen clearance (155, 156). PGE2 also impairs the *S. pneumoniae* intracellular killing (ICK) by AMs via inhibiting the hydrogen peroxide (H_2_O_2_) production (157). IL-18 produced by AMs protects against pneumonia and ALI associated with *S. pneumoniae* infection via enhancing the bacterial clearance (158). However, IL-18 proves detrimental to *P. aeruginosa*-induced pneumonia and enhances its invasiveness to cause sepsis and ALI (159). Thus, type (Gram-positive or Gram-negative) of bacterial pathogens also determines the macrophage-mediated immune response, including the protective or detrimental action of IL-18 released.

The transition of M1 to M2 macrophages during the late stages of pneumonia mediates the inflammation resolution via producing IL-4 and IL-13, which promote STAT6 activation (160). Also, pulmonary macrophages secrete TNF-α stimulated gene-6 (TSG-6), which helps in the ALI resolution via promoting the M1 to M2 macrophage transition However, the efferocytosis of neutrophils by AMs during later stages of pneumonia helps in the resolution of lung inflammation due to expression of growth arrest-specific 6 (Gas6), a member of vitamin K-dependent family of proteins, which binds to its receptors Tyro3, Axl and Mer (TAM), or Mertk (148, 160). The Mertk activation causes ERK-mediated sarcoplasmic/endoplasmic reticulum calcium ATPase 2 (SERCA2) expression to decrease the cytosolic Ca2^+^ levels, which suppresses the calcium//calmodulin-dependent protein kinase II (148). This process decreases the mitogen activating protein kinase (MAPK) and MK2 kinase activity to increase the abundance of non-phosphorylated cytosolic lipoxygenase (LOX), called 5-LOX, to enhance the production of specialized pro-resolving mediators (SPMs) mediating inflammation resolution (148). Thus, TSG-6 activates STAT6 to induce Gas6 expression in AMs for the ALI resolution during pneumonia.

Lipoxin A4 release by pulmonary endothelial cells, immigrated neutrophils, and pulmonary macrophages at later stages of pneumonia, inhibits neutrophil infiltration, promotes the efferocytosis of dead neutrophils by serving as a proapoptotic signal through downregulating Mac-1 (a β2 integrin) expression, to induce the pulmonary inflammation resolution (161–163). The lipoxin A4-induced neutrophil apoptosis involves the myeloperoxidase (MPO)-induced extracellular signal-regulated kinase (Erk) and Akt-mediated Bcl2-associated agonist of cell death (Bad) phosphorylation along with reducing the antiapoptotic protein myeloid cell leukemia-1 (Mcl-1) expression, which aggravates the mitochondrial dysfunction. This is because Mcl-1 promotes neutrophil survival through heterodimerization and neutralization of Bcl-2 interacting protein (Bim) or Bcl-2 homologous antagonist/killer (Bak) in the mitochondrial outer membrane (162, 164, 165). Lipoxin A4 also enhances the pathogen (*E. coli*) clearance by pulmonary macrophages through inducing the AMP expression (161). The Mac-1 binding to its ligands (ICAM-1, FBG, and MPO) suppresses the apoptosis (163). However, the Mac-1-dependent phagocytosis of complement-opsonized pathogens triggers rapid neutrophil apoptosis that depends on NADPH oxidase-generated reactive oxygen species (ROS) and caspase (CASP) activation (166). Lipoxin A_4_ also inhibits the CXCL8 or IL-8 release from pulmonary macrophages (167). Furthermore, Lipoxin A4 agonist, BML-111 induces autophagy in pulmonary macrophages through suppressing MAPK 1 and 8 signaling. The autophagy of pulmonary macrophages protects against ALI during Gram-negative bacterial pneumonia (168). Lipoxin A4-dependent autophagy among alveolar macrophages during pneumonia occurs independently of mTOR signaling. Hence pulmonary AMs play a crucial role in the induction of protective inflammatory immune during pneumonia and later on in the resolution of the inflammation.

This resolution process occurs at the expense of local pulmonary innate immunity comprising AMs (suppressing phagocytosis) to predisposes the recovering host to severe secondary pneumonia (169). This defective phagocytic function of AMs from pneumonia recovering animals stays for at least 28 days. Even the AMs transplanted intratracheally from normal mice to pneumonia recovered mice become paralyzed AMs, indicating the presence of long term inflammatory innate immune response suppression to make sure the complete resolution of the pulmonary inflammation (169). However, regulatory T cells (T_regs_), cytokines (TGF-β1 and TNF-α), and DAMPs (HMGB1) do not play a significant role in the induction of paralyzed AMs during resolution of pulmonary inflammation following pneumonia. Also, these paralyzed AMs are not metabolically exhausted as they produce more lactate as compared to the control AMs and produce the same amount of TNF-α upon LPS challenge. Of note, the process of macrophage renewal in mice recovering from pneumonia is similar to normal mice. Thus, AMs of mice recovering from pneumonia are defective in phagocytosis and are unable to clear bacterial pathogens efficiently, increasing their susceptibility to secondary pneumonia. However, these defective or paralyzed AMs are derived from the local pulmonary macrophages in response to the increased expression of signal regulatory protein α (SIRPα), a regulator of tyrosine kinase-coupled signaling processes (phagocytosis) (170–172).

SIRPα increases during the resolution phase in response to the increased surfactant protein-D (SP-D) level. SP-D is an agonist for SIRPα and induces the immunosuppressive environment to produce trained but paralyzed AMs, which stay for weeks after infection to make sure the complete resolution of the inflammation. The increased Sirpα expression upregulates the Mir142 (a micro RNA regulating gene expression in mononuclear phagocytes) expression but down-regulates Setdb2 gene (encoding a histone methyltransferase, which controls the chemokine response during viral pneumonia) (173, 174). The Sedtb2 down-regulation may prevent neutrophil infiltration during the resolution phase to dampen the pneumonia-induced ALI, as indicated previously (173). Also, the Sedtb2 down-regulation alter the pro-inflammatory phenotype of macrophages to a reparative phenotype (175). Mir142 is also shown to regulate immunometabolic reprogramming and favors glycolysis via regulating fatty acid oxidation (FAO) through directly targeting carnitine palmitoyltransferase −1a (CPT1a), a key regulator of the FA pathway (176). Thus, pulmonary macrophages play a crucial role in the resolution of ALI associated with pneumonia.

#### The Interaction Between PECs and AMs During Pneumonia and Associated ALI

The AMs highly express CD200R (an OX2 glycoprotein of the superfamily of immunoglobulins) on their surface and its levels are maintained by epithelial expression of IL-10 and TGF-β (177). The PECs express ligand for CD200R called, CD200 on their apical side. The CD200R-CD200 interaction on AMs inhibits their pro-inflammatory action during pneumonia that prevents the induction of ALI (177). The CD200-CD200R interaction increases AAMs or M2a phenotype via cAMP-response element-binding protein-C/EBP-beta signaling and upregulates TGF-β expression (178). Also, M2a (anti-inflammatory or regulatory) macrophages generated in the presence of IL-4 and IL-13 also express CD200R in humans (179, 180). The CD200-CD200R interaction inhibits the downstream signaling pathway comprising of the ERK1/2 signaling pathway required for macrophage activation downstream of IFN-γ signaling through Janus-associated kinase (JAK)/STAT-1 activation (181). The CD200^−/−^ mice develop ALI during pneumonia due to the increased pro-inflammatory function of macrophages and a decrease in the resolution of inflammation (177). Also, the AMs attached to the alveolar wall form connexin 43 (Cx43)-containing gap junction channels with the airway epithelium during bacterial pneumonia and intercommunicate through synchronized Ca^2+^ waves, through utilizing the epithelium as the conducting pathway (182, 183). This interaction further supports the anti-inflammatory role of PEC-AM interaction. As mice with AM-specific knockout of Connexin-43 show an increased neutrophil infiltration into the pulmonary alveoli and increased pro-inflammatory cytokine levels in BALF during Gram-negative bacterial (*P. aeruginosa*) pneumonia (182, 183). Thus, the interaction between PECs and AMs controls the inflammatory outcome of the pulmonary infections, including pneumonia leading to the development of ALI and its resolution.

## Pulmonary Innate Immune Response During Bacterial Sepsis

**Figure 3:**
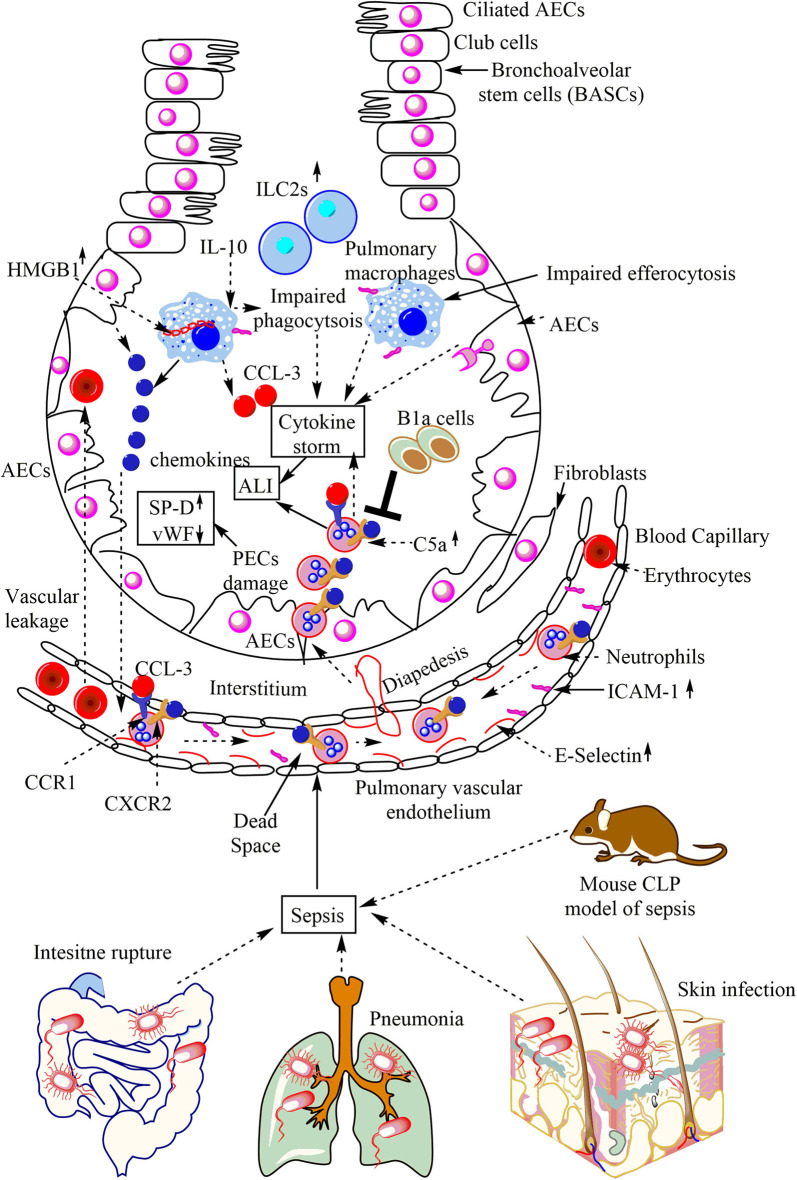
Overview of sepsis-induced ALI. Local infections of the skin (*S. aureus*), lungs (pneumonia), and intestinal commensal bacteria leak into the blood may lead to sepsis development. Sepsis leads to the neutrophil and monocyte infiltration in the lung alveoli via pulmonary transendothelial migration due to the profound release of the pro-inflammatory mediators (cytokine storm) damaging endothelial monolayer and inducing endothelial vascular leakage. These neutrophils and monocytes reach into the lung alveoli through crossing the pulmonary epithelial layer due to damage of PECs (AECs and BECs). These PECs express C5aR and C3aR receptors. The profound release of complement component C5a during sepsis induces the inflammatory damage, and the death of PECs during sepsis causes sepsis-associated ALI. The PECs death/damage increases the SP-D, but Vwf levels decrease. The increase in the IL-10 levels in the lungs at later stages of sepsis impairs the bactericidal action of AMs along with inducing a defective efferocytosis. The defective efferocytosis among AMs further increases the ALI. The necrotic death of AMs (indicated by the cytosolic HMG-B1) at later stages of sepsis further aggravates the ALI. The neutrophils infiltrated into the lung alveoli during sepsis are apoptosis-resistant and aggravate the ALI due to their increased pro-inflammatory action on lung tissues. B1a cells inhibit neutrophil infiltration and, thus, the sepsis-induced ALI. The increase in pulmonary ILC2s also occurs.

Sepsis leads to the pulmonary inflammation that does not resolve and leads to the development of ALI or ARDS, causing irreversible damage to the lungs (12). Earlier studies have shown that the sepsis is responsible for more than 210,000 cases of ALI/ARDS in the US alone/annually, causing over 74,500 deaths (184, 185). The sepsis-associated ALI//ARDS has a higher mortality rate as compared to the ALI occurring due to other causes (186). The sepsis-associated ALI/ARDS may initiate on any side, including direct lung injury due to the pulmonary epithelial damage or indirect damage comprising the endothelial cell damage (187, 188). The neutrophil infiltration plays a crucial event in this outcome, and the recruitment of neutrophils into the lungs depends on the expression of E-selectin [CD62E or endothelial-leukocyte adhesion molecule 1 (ELAM-1), or leukocyte-endothelial cell adhesion molecule-2 (LECMA-2)]. E-selectin does not express on unstimulated endothelium, but its expression increases on pulmonary vascular endothelium due to the impact of pro-inflammatory cytokines and induces neutrophil infiltration in sepsis-induced ALI (Figure 3) (189).

The immunohistochemical analysis has shown an increased expression of CD62E or E-selectin in the pulmonary microvasculature in sepsis-associated fatalities. The pulmonary intravascular, interstitial and intra-alveolar leukocytes strongly express very late antigen-4 or VLA-4 (CD49d/CD29) or α4β1integrin in sepsis-associated casualties. The ICAM-1 (CD54) is highly expressed on the pulmonary endothelial cells, pulmonary macrophages, and lymphocytes in sepsis-associated fatalities (Figure 3). The pulmonary epithelial damage during ALI/ARDS seen in patients with pneumonia-associated sepsis may be indicated by an elevation of surfactant protein-D (SP-D). However, but these patients have low levels of von Willebrand factor (vWF) and IL-6, and IL-8, which are the markers of endothelial damage (Figure 3) (190). The pulmonary epithelial damage seen during direct sepsis-associated ALI is more severe as compared to the indirect non-pneumonia-mediated sepsis (190). However, the damage to the endothelium during direct sepsis-associated ALI is less severe. The pulmonary B1a cells exert a protective role in cecal-ligation and puncture (CLP)-induced sepsis via inhibiting neutrophil infiltration and MPO production in the lungs (Figure 3) (191). The CXCR2^−/−^ mice exposed to peritoneal sepsis show a decreased pulmonary damage due to the low neutrophil infiltration in the lungs, and the increased CXCL10 expression in the peritoneum (192). CCL-3 or macrophage inflammatory protein-1α (MIP-1α) also mediates sepsis-induced ALI via promoting neutrophil infiltration, pulmonary vascular leakage, and early mortality (Figure 3) (193). The following sections highlight the pulmonary innate immune response during sepsis-induced ALI//ARDS.

### PECs During Sepsis and Associated ALI

The generation of pro-inflammatory molecules (cytokines, ROS, and RNS) and hypoxia damage the pulmonary epithelial barrier during sepsis-induced ALI (Figure 2) (194, 195). This damage to the pulmonary epithelium alters its barrier function and induces the fluid and protein leakage into the alveolar space. The injury to both type I and II AECs during sepsis can easily be assessed in both plasma and pulmonary lavage fluid by the presence of several biomarkers as described previously (194, 196). The pulmonary epithelial damage and increase in its permeability during sepsis involve the change in actin organization (197). The PECs damage due to actin reorganization during sepsis does not include MAPK signaling or the alterations in the tight junction (TJ) proteins. The PECs (BECs and AECs) of the septic lung show an increased αvβ3 integrin, but its inhibition during sepsis-associated ALI needs to study as it may increase the endothelial permeability and thus the sepsis-associated ALI (198, 199). The later (proliferative or fibroproliferative stage observed during the second week of sepsis) stages of ALI during sepsis involves the transformation of the damaged epithelial cells to fibroblast-like cells (epithelial-mesenchymal transition), which requires mitochondrial ROS and hypoxia-inducible factor-1α (HIF-1α) (200).

The PECs express a higher Fas level during sepsis-associated ALI, and the increased infiltration of FasL expressing inflammatory immune cells in the lungs occurs (201). The apoptotic death of PECs during non-pulmonary sepsis (sepsis originating outside the lungs or in the absence of pulmonary infection) involves Fas-FasL interaction, and the Fas inhibition protects their apoptotic cell death via diminishing lung tissue TNF-α, IL-6, IL-10, IFN-γ, IL-12, and CASP-3 activity (202). The BECs show an increased expression of both C3aR and C5aR during sepsis (203). An increased intrapulmonary or intra alveolar C5a level during sepsis may cause severe ALI via binding to the C5aR1 or C5aR, which induces an increased neutrophil infiltration into the septic lung and cytokine/chemokine storm (Figure 3) (204, 205). The infiltrated neutrophils in the lungs during ALI/ARDS have a distinctive phenotype and are resistant to apoptosis, and exhibit an enhanced phosphoinositide 3-kinase-dependent (PI3K)-dependent respiratory burst (206). A human study has also indicated the infiltration of less apoptotic neutrophils in the lungs of Sepsis-associated ALI/ARDS patients (207). Hence neutrophils migrated to the lungs during sepsis-associated ALI exert more damaging effects to the lungs as compared to bacterial pneumonia (Figure 3).

The apoptosis of neutrophils enhances the resolution of the inflammation that is lost in the sepsis-associated ALI. However, the cyclin-dependent kinase (CDK) inhibitor, called AT7519 enhances the apoptosis of infiltrated neutrophils during sepsis-associated ALI or ARDS, can be used as a mediator of initiating the resolution phase of inflammation during sepsis-associated ALI (208). Of note, mechanisms causing ALI and resolution of inflammation occur in parallel during sepsis-associated ALI/ARDS. The first resolution step involves the reestablishment of the alveolar-capillary barrier and the migration of AT-II epithelial cells to replace injured AT I epithelial cells, following the proliferation of tissue-resident progenitor cells (187). However, the uncontrolled inflammatory process causing severe ALI overpowers the resolution process, which causes irreversible damage during Gram-negative bacteria (*Klebsiella pneumoniae*)-induced sepsis as indicated by accumulation of the lungs with apoptosis-resistant neutrophils and elevation of pro-inflammatory cytokines (IL-1α, TNF-α) in BALF (12). Furthermore, keratinocyte growth factor (KGF) treatment induced the resolution in PECs *in vitro* and *in vivo* in mice, but it failed in phase II clinical trial and aggravated the ALI (209–211). Thus, due to severe PEC damage during sepsis-induced ALI, it is more damaging and irreversible as compared to the ALI observed during bacterial pneumonia only without the development of sepsis.

### ILCs During ALI/ARDS Observed During Sepsis

There is a doubt regarding the presence of ILC1s in naïve lungs or during homeostasis. However, their number increases during Haemophilus influenzea infection. It occurs due to the phenotypic change in lung-resident ILC2s in response to the downregulation of T1/ST2, GATA3, IL-5, and IL-13 expression (125). ILC2s represent the majority of ILCs in both mouse and human (30% of all ILCs in adult human lungs) lungs (212, 213). Although Lungs have a low number of ILC2s in the steady-state, and it increases only during pulmonary allergic diseases. Lungs have both NCR^+^ and NCR^−^ ILC3s during a steady state. Lin-CD127^+^RORγt^+^ILC3s comprise 30% of ILCs in mice, and the majority of them also co-express CCR6. These can be activated with IL-23 and IL-1β *in vitro* to produce IL-22 and IL-17A (135). The human lungs also have ILC3s (Lin^−^CD127^+^CRTh2^−^CD117^+^) expressing RORγt. During *S. pneumoniae* lung infection, the depletion of ILC3s protects the host from ALI due to inhibition of IL-22 and IL-17A production (135).

The systemic levels of ILCs (ILC1s and ILC3s) significantly decrease in patients with sepsis in comparison to the control group due to their increased apoptotic death (214). The HLA-DR expression increases in the ILCs of the septic patients without any effect on their capacity to produce TNF-α in response to the TLR agonists. The apoptotic cell death among ILCs (ILC1s, ILC2s, and ILC3s) occurs due to the increase in CASP3 level and activity within <24 h of sepsis diagnosis (214). However, no significant decrease in systemic ILC2s occurs during the early stages of sepsis despite the increase in CASP3 activity. It may be attributed to the sphingosine-1-phosphate (S1P)-dependent migration of ILC2s to distant organs, including lungs (215). The plasma S1P-1 level decreases with the severity of the sepsis (216). The ILC2 migration to the lungs in response to S1P occurs due to increased expression of S1P receptors (S1PRs, S1P1-SIP5) (217). A study in CLP-induced sepsis has shown the increase in ILC2s in the peritoneum and small intestine along with the increased IL-13 and IL-33 levels in the peritoneal lavage fluid (PLF) within 24 h post sepsis development (218).

Patients with sepsis show increased plasma IL-33 levels (218). Many investigators have suggested different roles of pulmonary ILC2s during sepsis, depending on the experimental model. For example, increased IL-33 levels (released by epithelial cells of the lungs) in CLP-induced sepsis in mice cause sepsis-induced ALI, and IL-33 inhibition causes a decrease in sepsis-associated ALI due to the decreased neutrophil and monocyte infiltration into the lungs (219). This IL-33 dependent ALI during sepsis also occurs via IL-5 upregulation in pulmonary ILC2s, and the IL-5 neutralization decreases the neutrophil infiltration, and ALI during sepsis (219). Thus, an increased activation of pulmonary ILC2s during CLP-induced sepsis may contribute to the sepsis-associated ALI. However, another study has shown the protective effect of the pulmonary ILC2s during sepsis-induced ALI via preventing the endothelial cell damage in response to the IL-33 released, which via binding to the ST2 receptor, mediates the ILC2 expansion (220).

The pulmonary ILC2s produce IL-9, which prevents CASP1 activation and the pyroptosis of pulmonary endothelial cells. It reduces the sepsis-associated ALI severity. The pulmonary ILC2s increase within the first 12 h of the sepsis development along with an increase in the peritoneal ILC2s (220). However, in another study, the ILC2s (as measured by the production of IL-5 and IL-13) pre-activation via intra-tracheal IL-33 administration before the lethal *S. aureus* sepsis protects the host from ALI and death via pulmonary eosinophilia induction, which clears the pathogen from the lungs and suppresses neutrophilia (221). However, without IL-33 pre-treatment, *S. aureus* is unable to induce ILC2 proliferation and function. Hence ILC2s play both beneficial and detrimental roles in ALI and sepsis-associated mortality depending on their activation stage. It will be interesting to investigate the impact of sepsis-associated ALI in people previously affected with parasitic infections causing a rise in pulmonary ILC2s and eosinophilia. Thus, pulmonary ILCs are crucial innate immune cells of the lungs, but their relevance to the sepsis-induced ALI/ARDS is a topic for the research and future immunomodulatory therapeutics. However, a decrease in the systemic ILC population is well-described even during the early phase of the sepsis.

### Alveolar Macrophages (AMs) and Sepsis-Induced ALI//ARDS

The pro-inflammatory mediators released from AMs play a crucial role in the sepsis-induced ALI via inducing neutrophil infiltration into the lungs (Figure 3). The interstitial-to-vascular chemotactic gradient establishment facilitates the emigration of the vascular neutrophils in the lung alveoli (Figure 3) (222, 223). The bacterial peritonitis-induced sepsis activates AMs and neutrophil infiltration in the lung alveoli (Figure 3). The neutrophil infiltration in the lung alveoli occurs via pulmonary transendothelium in response to the AM activation during sepsis (Figure 3) (224). The NADPH oxidase activation in the pulmonary endothelium generates superoxide anion in response to the AM activation that plays a crucial role in the transendothelial neutrophil migration during sepsis-associated ALI (224). These neutrophils are less prone to apoptotic cell death and play a significant role in the sepsis-induced severe ALI (Figure 3). Furthermore, these infiltrated neutrophils block pulmonary microcirculation due to their prolonged entrapment in the capillaries inducing the dead space, which further aggravates the sepsis-induced ALI (Figure 3) (225). These neutrophils express Mac-1 (CD11b/CD18), and the Mac-1 inhibitor decreases the incidence of disturbing pulmonary microcirculation and the sepsis-induced ALI. Also, the impaired phagocytic activity of AMs during late stages in response to the released IL-10 during abdominal sepsis further enhances the incidence and the severity of sepsis-induced ALI (Figure 3) (226).

The impaired efferocytosis by AMs during the late stages of sepsis further increases the severity of ALI/ARDS due to the accumulation of dead neutrophils and other pulmonary cells (Figure 3) (227). However, the IFN-β treatment reverses the impaired AM function in response to the IL-10 at the late stage of sepsis and decreases the severity of sepsis-associated ALI/ARDS and the associated mortality (228). The HMG-B1 release in the cytosol of AMs during the late sepsis indicates their necrotic cell death, which further increases the ALI severity (Figure 3) (229). The macrophages endocytose HMG-B1 during sepsis (230). The HMG-B1 promotes pyroptosis of macrophages and endothelial cells by delivering the LPS via the receptor for advanced glycation end products (RAGE) into the cytosol, which destabilizes phagolysosome and induces CASP11 activation during lethal sepsis (231).

The CASP11 is the important inflammasome component, and HMG-B1 is known to activate NLRP3 inflammasome and the IL-1β release. The CASP11 activation causes pyroptosis via cleaving gasdermin D (GSDMD) into amino-terminal GSDMD (N-GSDMD) and carboxy-terminal GSDMD (C-GSDMD) (232, 233). The N-GSDMD is responsible for the pyroptosis. The increased lipid peroxidation (LPO) in the sepsis-associated ALI has been observed (12). This increased LPO further activates CASP11, and thus, the GSDMD to cause the pyroptosis of AMs and infiltrated monocytes and macrophages in a phospholipase C gamma 1 (PLCG1)-dependent manner (234). Also, the inflammatory IL-1β reduces the cyclic adenosine monophosphate (cAMP) and transcription factor cAMP response element-binding (CREB) in lung endothelial cells (235). This CREB blockage inhibits the VE-cadherin transcription, which induces pulmonary vascular endothelial damage to aggravate pulmonary vascular leakage and sepsis-associated ALI. Also, the treatment with rolipram (a drug inhibiting the type 4 cyclic nucleotide phosphodiesterase–mediated (PDE4-mediated) hydrolysis of cAMP) prevents sepsis-induced pulmonary vascular injury and thus the ALI via preserving the CREB-mediated VE-cadherin expression (235). Of note, the deficiency of neutrophils before sepsis also impairs the monocyte and macrophage infiltration in the lungs during both early and late stages and thus inflammatory process (236).

The iNOS induction in AMs during sepsis also causes protein leakage in the lungs and sepsis-induced ALI. The AM depletion attenuates the sepsis-induced increase in pulmonary microvascular protein leak and MPO activity that depends on the activation of iNOS (237). The increased nitric oxide (NO^.^) and MPO levels in BALF and lung homogenate of mice subjected to *K. pneumoniae* B5055-induced sepsis has been reported on all days in an experimental study (12). Furthermore, microRNA-199a (miR-199a) upregulation in AMs during Gram-negative bacterial sepsis also aggravates the sepsis-induced ALI, which can be prevented by the activation of sirtuin 1 [SIRT1 (Silent information regulator 2 homolog 1), an NAD^+^-dependent class III protein deacetylase or histone deacetylase regulating cell growth, differentiation, stress resistance, oxidative damage, and metabolism] (238, 239). The induction of miR-199a in AMs during sepsis increases the release of pro-inflammatory cytokines (IL-1β, IL-6, and TNF-α), the MPO activity, ALI, and the high levels of CASP3, Bax and lowers the Bcl-2 levels (238). The miR-199a inhibition during sepsis decreases the release of pro-inflammatory cytokines from AMs, MPO activity, the incidence of vascular leakage from pulmonary endothelium. The SIRT1 activation during sepsis also prevents the sepsis-induced ALI via inhibiting the NLRP3 inflammasomes in AMs and pulmonary vascular endothelial cells, which prevents the release of pro-inflammatory mediators (ICAM-1 and HMG-B1), disruption of tight and adherens junctions as indicated by the reduced lung claudin-1 and vascular endothelial-cadherin expression (240, 241).

The sepsis-associated altered AM function predisposes these mice to severe pulmonary infections and increases their mortality when challenged with Gram-negative bacteria (*P. aeruginosa*) due to the IL-1 receptor-associated kinase–M (IRAK-M) upregulation, which causes sepsis-associated immunosuppression at later stages (242). IRAK-M-mediated impaired TNF-α and iNOS expression in AMs is associated with the reduced acetylation and methylation of specific histones (AcH4 and H3K4me3) and reduced binding of RNA polymerase II to the promoters of these genes (243). However, the TLR2 and TLR4 levels remain the same in septic lungs as compared to the control group (242). Of note, diabetic rats show milder sepsis-associated ALI due to the impaired activation of nuclear factor kappa-light-chain-enhancer of activated B cells (NF-κB), increased suppressor of cytokine signaling 1 (SOCS1), and decreased MyD88 mRNA, and thus the decreased MyD88 downstream signaling in response to the TLR stimulation on AMs (244). The decreased AM activation in diabetic rats inhibits neutrophil infiltration, cyclo-oxygenase II (COX-II) expression and activity, and the pulmonary edema. The low incidence of sepsis-induced ALI has been also observed in patients with diabetes developing sepsis (245–248). However, a recent clinical study indicates that diabetes does not have any impact on sepsis-associated mortality and the 60-days mortality of ALI/ARDS (249). Diabetes may reduce the incidence of the sepsis-induce ALI/ARDS but not the associated mortality. Further studies in the direction and the establishment of AMs role in clinical patients of sepsis with diabetes about the ALI may prove helpful in a patient-specific therapeutic approach.

Hence sepsis leads to the severe ALI as compared to the ALI seen in pneumonia only patients. Furthermore, sepsis causes prolonged immunosuppressive stage in the lungs, which increases the chances of developing severe secondary pulmonary infections (hospital-acquired or community-acquired). For example, the pulmonary alveolar macrophages decrease in number in patients recovered from sepsis and show defective phagocytic function against bacterial pathogens (*E. coli* and *S. aureus*), which are frequently responsible for hospital-acquired pneumonia (169). This defective number of pulmonary alveolar macrophages stays at least for 6 months. These clinical findings (severely compromised phagocytic activity of AMs) have further been confirmed in mice subjected to secondary pneumonia caused by *E. coli* or *S. aureus* (169). These macrophages from patients recovering from sepsis also showed increased SIRPα expression. However, the outcome of sepsis-associated ALI may also depend on several other chronic inflammatory conditions, including type 2 diabetes mellitus (T2DM). Further studies are urgently required in the field due to the high mortality of sepsis patients due to the Sepsis-induced ALI/ARDS.

## Conclusion

Pneumonia and sepsis, both are associated with the onset of ALI/ARDS. However, the pneumonia-associated ALI is less severe and often resolves once the infection has cleared. But this resolution of ALI during sepsis has not been observed. However, the pneumonia-associated ALI resolution leaves a long-lasting impact on the host immune response to future infection. This resolution of ALI involves the transforming growth factor-β (TGF-β) generation and the activation of pulmonary regulatory T cells (Tregs) inducing the immunosuppressive environment in the lungs (169). It causes the induction of paralyzed pulmonary macrophages and DCs, which are defective in the phagocytosis of the pathogen but further secrete TGF-β responsible for the Tregs accumulation. These paralyzed DCs express an increased amount of transcription repressor called B lymphocyte-induced maturation protein-1 (Blimp-1) but a lower amount of interferon regulatory factor 4 (IRF4). Blimp-1 is essential for tolerogenic DCs. Thus, the increased expression of Blimp-1 induces a tolerogenic phenotype of DCs. Whereas, IRF-4 is for expressing the molecules required for the antigen presentation, and its lower level in paralyzed DCs decrease their antigen presentation potential.

The immunosuppressive environment in the lung following the resolution of ALI-associated with pneumonia further disposes the host to acquire secondary pulmonary infection. However, this can be avoided by following the instruction, like keeping the recovered patient in the pathogen-free environment and keeping him/her on the immune-boosting diet. For example, the study has shown that these paralyzed macrophages and DCs generated following the resolution of pneumonia-associated ALI remains active for at least 21 days post clearance of the pathogen (169). Hence the impairment in the expression of Blimp-1 and IRF-4 in other immune cells, including PECs, T, and B cells post, pneumonia should also be studied to explore the unknowns associated with the cost of resolution of ALI associated with pneumonia and the patients recovered from sepsis. ALI during sepsis proves detrimental to the host. Even the neutrophils and monocytes infiltrated into the septic lungs show the apoptosis-resistant phenotype that proves harmful to the host by further aggravating the sepsis-induced ALI. Thus, therapeutics able to induce their phagocytosis later in the sepsis will prove beneficial to prevent the sepsis-induced ALI. Keeping in mind the difference in the action of the pulmonary innate immune response during sepsis and pneumonia-induced ALI, different therapeutics should be designed as the drug or molecule worth for one may not be useful for the other. Future studies are required in the direction to prevent the sepsis or pneumonia-induced ALI by studying the pulmonary, innate immunity. For example, the discovery of ILCs in the lungs and further research in their functional role in pneumonia and sepsis-induced ALI has changed their pathogenesis and opened the door to design better and new therapeutics, including the vaccines for pneumonia.

## Author Contributions

VK has developed the idea, searched the literature, compiled, and wrote the article.

## Conflict of Interest

The author declares that the research was conducted in the absence of any commercial or financial relationships that could be construed as a potential conflict of interest.
